# Dissecting the difference: Left ventricular aneurysm versus pseudoaneurysm

**DOI:** 10.21542/gcsp.2025.29

**Published:** 2025-06-30

**Authors:** Muhammad Salman Sabri, Kyra Herman, Hussam Al Hennawi, Alexander Shpilman, James L. West

**Affiliations:** 1 Department of Internal Medicine, Jefferson Abington Hospital, Abington, PA, USA; 2 Department of Cardiology, Jefferson Abington Hospital, Abington, PA, USA; 3 Department of Cardiothoracic Surgery, Jefferson Abington Hospital, Abington, PA, USA

## Abstract

Left ventricular aneurysms (LVAs) and pseudoaneurysms (LVPs) are common complications following acute myocardial infarction (AMI), predominantly involving the anterior wall due to complete occlusion of the left anterior descending (LAD) artery. Both conditions are associated with heart failure, arrhythmias, and thromboembolic events. However, differentiating between LVA and LVP is crucial, as LVPs carry a higher rupture risk and require surgical management. We present a case of LVP, diagnosed using cardiac magnetic resonance imaging (CMRI), in a patient with chronic total occlusion of the LAD, which was managed surgically. This case emphasizes the critical role of imaging in guiding appropriate treatment decisions.

## Background

Patients with AMI are at an elevated risk of LVA and LVP, particularly apical aneurysms. Historically, the incidence of LVAs was 11% before the widespread use of modern reperfusion techniques. Following the adoption of reperfusion strategies, such as PCI, the incidence has become more variable, ranging from 3.5% to 9.4%. LVPs are rare, with an incidence of less than 0.1%, but are associated with a high risk of rupture and mortality.

## Case report

A 64-year-old sedentary male with no known prior cardiac events and a history of type 2 diabetes, hypertension, hyperlipidemia, and 38 pack/year tobacco use presented after a witnessed out-of-hospital cardiac arrest. His wife heard him gasping and found him unconscious and slumped in the chair. Emergency medical services were called and he was defibrillated for VF and intubated prior to arrival.

An ECG showed ST elevations in V4–V6 as well as Q waves in the anterior wall leads, leading to urgent cardiac catheterization, which revealed a chronic total occlusion of the LAD and 90% distal LCX occlusion. The LAD was completely occluded just beyond the first septal perforator, consistent with chronic total occlusion. Multiple soft wires were initially used to cross the LAD lesion, but all attempts were unsuccessful. Subsequently, using an over-the-wire system, several wires, including a Pilot 50 and Miracle Brothers wire, were directed at the occlusion; however, these also failed to cross the lesion. In the AV groove, the circumflex artery exhibited 90% stenosis just before the origin of a large second obtuse marginal branch. Balloon dilation and stenting of the LCX lesion were successfully performed. Post-procedure, TTE revealed worsening LV function with an EF of 10% and global hypokinesis. The patient initially required pressor support due to cardiogenic shock but improved after undergoing angioplasty. He was initiated on guideline-directed medical therapy (GDMT) and discharged with a LifeVest.

**Figure 1.**
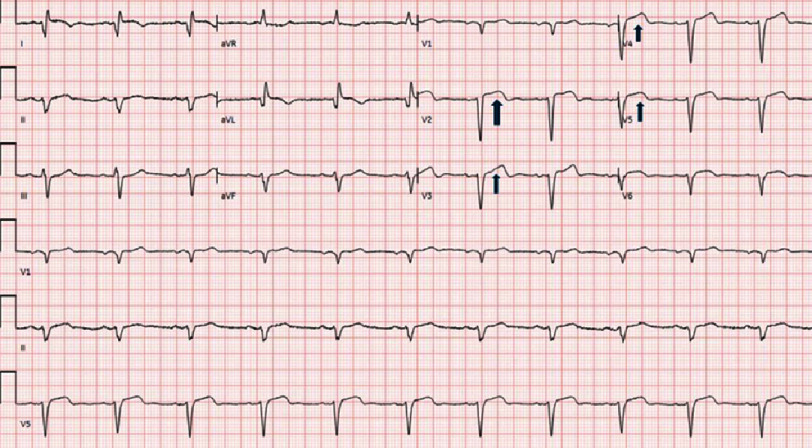
The electrocardiogram displays persistent ST-segment elevation in the precordial leads (indicated by arrows), along with Q-waves in the lateral and anterior leads and poor R-wave progression.

**Figure 2.**
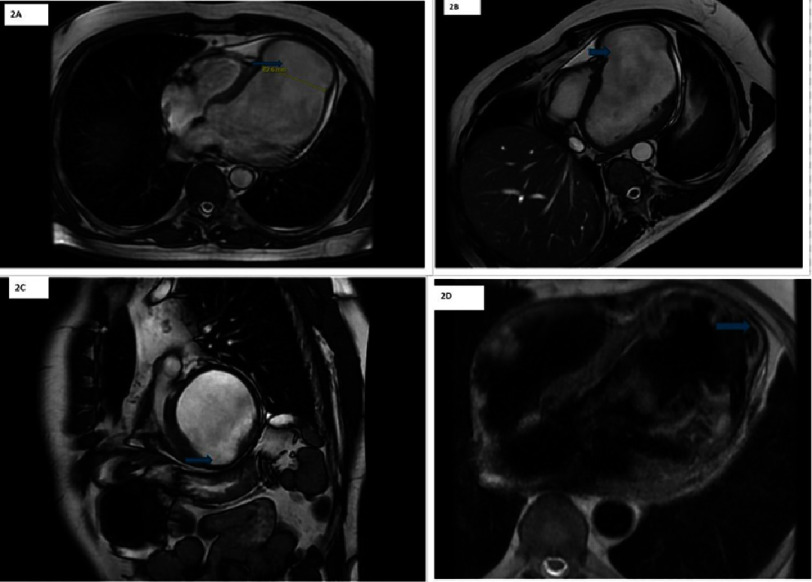
Cardiac MRI showed an aneurysm with a maximal diameter of 7 cm (2A) and a neck diameter of 6.5 cm (2B and 2C), suggesting a left ventricular pseudoaneurysm. Layering of thrombus within the aneurysm is observed (2D), raising concerns for pseudoaneurysm formation.

**Figure 3.**
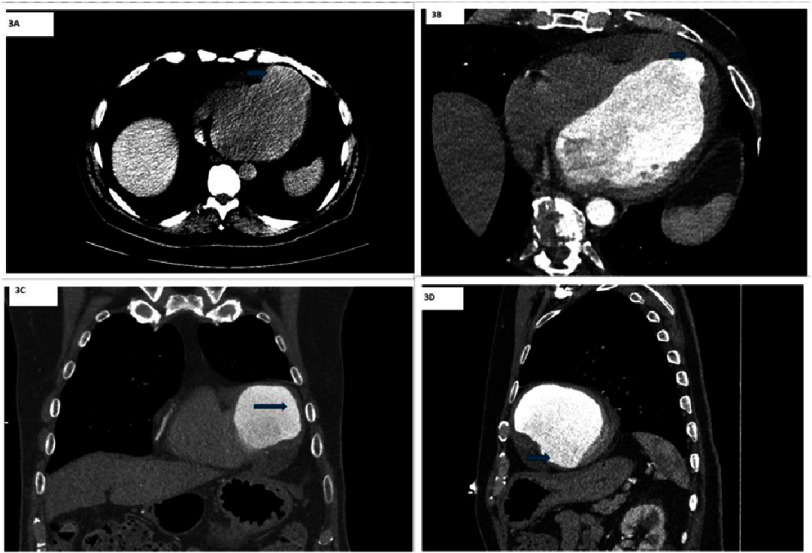
Contrast-enhanced CT of the chest and aorta reveals focal dilatation at the left ventricular apex (3A, 3B, 3C, and 3D) with a narrow base and abrupt transition, raising suspicion for a left ventricular pseudoaneurysm.

One month after discharge, he had a cardiology follow-up appointment, during which he had no acute complaints. Four months after his initial MI, he saw a heart failure specialist, where a repeat ECG showed persistent ST-segment elevation in the precordial leads, concerning for LVA or LVP [Figure 1]. Cardiac magnetic resonance imaging (CMRI) [Figure 2] demonstrated significant myocardial damage in the LAD territory and a 7 cm left ventricular apical outpouching with a neck diameter of 6.5 cm and a thrombus lining its wall, likely a pseudoaneurysm. The LV ejection fraction was severely reduced to 6%, and a small pericardial effusion was noted. CT chest and aorta with contrast [Figure 3] was performed to better visualize his anatomy for surgical planning and confirmed a dilated LV and ventricular outpouching consistent with LVA or LVP.

**Figure 4.**
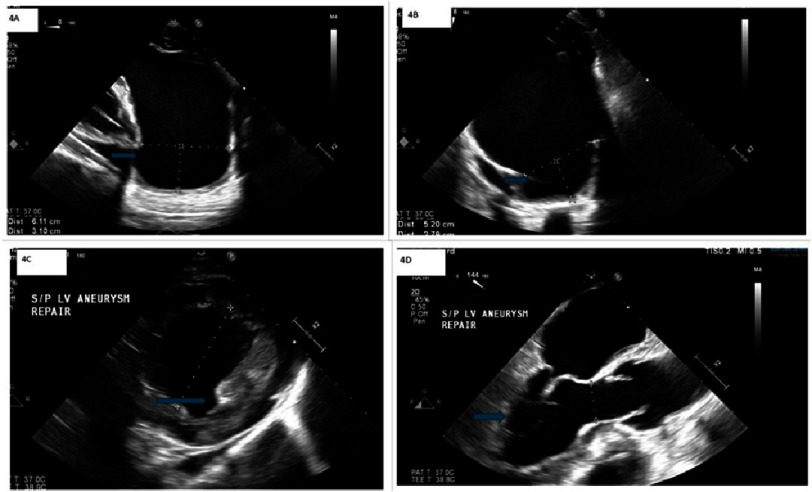
Intraoperative transesophageal echocardiography (TEE) shows a 6.3 cm left ventricular apical aneurysm (4A, 4B), with hypokinesis of the basal wall and akinesis of the remaining walls, resulting in an ejection fraction of 5 to 10 percent. Post-surgical repair TEE (4C, 4D) demonstrates normal left ventricular size, with moderate hypokinesis of the lateral and inferior walls extending to the papillary area, and akinesis of the remaining walls. The ejection fraction is now measured at 15–20 percent.

The patient was optimized with an intra-aortic balloon pump (IABP) for LV aneurysm repair. The patient underwent LV aneurysm repair three weeks after the diagnosis of LVA/LVP. Intraoperative TEE [Figure 4] demonstrated a 6.3 cm LV apical aneurysm and EF of 5%. After sternotomy, the LV aneurysm was carefully dissected from the pericardium. Pericardial adhesions made it difficult to distinguish between a contained left ventricular free wall rupture and a left ventricular pseudoaneurysm. The LV was opened through the aneurysm, and a 3 cm defect was repaired using a Dacron patch, which is known for its biocompatibility, durability, and structural integrity. During recovery, he was noted to have expected postoperative anemia, for which he received blood transfusions. His hemoglobin levels stabilized, and he was discharged.

One week after the operation, a single-chamber ICD was implanted, and TTE showed moderate hypokinesis of the lateral and inferior walls with akinesis of the remaining walls. The ejection fraction improved to 15–20 percent. His postoperative recovery was complicated, and he was hospitalized for three days for symptomatic anemia 11 days after LVA/LVP repair. A CT of the chest showed a small pericardial effusion and small ill-defined mediastinal fluid collection, which was evaluated by a surgical team to be stable postoperative changes and not the cause of his anemia. Repeat TTE revealed a stable EF. His hemoglobin levels stabilized, and he was discharged.

## Discussion

LVA typically develops following a period of severe ischemia and inflammation, and the subsequent replacement of myocardial tissue with fibrous tissue and scarring. This leads to an area of thin and dyskinetic or akinetic LV. In contrast, LVP results from the rupture of the LV free wall, which is contained within the adjacent pericardium^1^. LVA usually occurs in the anterior wall in the setting of LAD territory MI but is less common in the inferior and lateral wall in the setting of dual blood supply from LCA and RCA^1^. In contrast, LVP can occur in the lateral, inferior, or posterior LV walls^1^.

The primary risk factor for the development of LVA and LVP is MI, particularly STEMI involving the anterior wall. It is associated with total occlusion of the left main or left LAD and usually presents 1 to 4 weeks after MI. Other contributing factors include delayed or inadequate reperfusion during MI and the absence of collateral circulation beyond the site of blockage^4^.

Non-ischemic risk factors for LVA include prior cardiac surgery, hypertrophic cardiomyopathy, Chagas disease, and sarcoidosis. In contrast, the risk factors for LVP are primarily associated with prior cardiac surgery and trauma^4^. LVP located on the posterior or lateral wall, with a narrow neck and associated symptoms such as heart failure, chest pain, or arrhythmias, carry a higher risk of rupture^6^.

Patients with LVA may be asymptomatic or present with a range of symptoms, including chest pain, dyspnea, dizziness, hypotension, heart failure symptoms, presyncope, or syncope, often due to arrhythmias^1^. LVA is frequently associated with mural thrombus formation, which can lead to systemic thromboembolic events^1^. Additionally, these patients are at an elevated risk of sudden cardiac death, particularly in the context of life-threatening arrhythmias^1^. The physical examination can be remarkable for diffuse apical impulse, S3 or S4 gallop, and systolic murmur at the apex from mitral regurgitation^1, 2^.

ECG typically demonstrates signs of an anterior wall MI with persistent ST elevation in the precordial leads^1^. TTE is generally the first imaging modality used to diagnose LVA or LVP in patients with risk factors and relevant clinical signs and symptoms^1^. Transesophageal echocardiography (TEE) can be considered when TTE results are inconclusive. TTE and TEE are both influenced by image quality and operator variability, although TEE typically has less variability owing to its superior image resolution. However, it remains operator-dependent. CMRI is the gold standard for diagnosing LVA or LVP^1^. In cases where CMRI is contraindicated, such as in patients with pacemakers or metallic implants, coronary CTA and contrast ventriculography serve as alternative diagnostic options^1^.

Imaging differentiation between LVP and LVA is primarily based on the characteristics of the aneurysmal neck, with particular attention to comparing the neck diameter, the maximal aneurysmal diameter, and the flow dynamics within the cavity. LVA typically exhibits a broad neck, a large dyskinetic or akinetic myocardium area during diastole, and abnormal myocardial deformation during systole without disruption of the myocardial lining^3^. In contrast, LVPs are characterized by a narrower neck relative to the maximal diameter and an abrupt transition from myocardium to the pericardium. They are often associated with turbulent blood flow^3^. CMRI is particularly valuable in distinguishing between the pericardium, myocardium, and thrombus, providing superior tissue characterization to aid in accurate diagnosis^3^. Diffused pericardial enhancement on CMRI can help diagnose LVP^4^. Up to 50% of patients with LVA have LV mural thrombus. CMRI can identify thrombus through low signal intensity on delayed gadolinium enhancement sequences. Additionally, multi-detector computed tomography (MDCT) can detect thrombus as a low-attenuation mass next to thinned myocardial tissue.

The management of LVA requires a multifaceted approach. This includes reperfusion therapy if myocardial ischemia is present and GDMT for patients with reduced ejection fraction and ischemic cardiomyopathy. The approach typically involves beta-blockers, renin-angiotensin-aldosterone system inhibitors, sodium-glucose co-transporter two inhibitors, and, when indicated, ICD or cardiac resynchronization therapy.

Patients with LVA should be routinely screened for LV thrombus. Anticoagulation therapy should be initiated if a thrombus is detected to reduce the risk of thromboembolic events. Surgical intervention is indicated for selected patients, particularly those undergoing concomitant cardiac surgery (e.g., coronary artery bypass grafting or valve replacement).

Surgery is also recommended for patients with progressive heart failure, refractory arrhythmias, or embolic events despite optimal GDMT and anticoagulation therapy^5^. Surgical repair is typically performed through aneurysmectomy, which may involve linear repair or left ventricular reconstruction using a pericardial patch. The latter approach has demonstrated more favorable long-term outcomes by restoring the original geometry of the LV. Prior case reports have also mentioned better outcomes from surgical repair^7^, especially in high-risk patients.

Patients with an LVP typically require surgical intervention due to the high risk of rupture, which is associated with a mortality rate of approximately 50%.

### What have we learned?

• LVA and LVP remain high-risk complications of acute myocardial infarction despite advances in reperfusion strategies, especially in anterior STEMI.
• Multimodal imaging—including CMRI and CT—is essential for accurate differentiation between LVA and LVP, which directly impacts clinical management and surgical planning.
• Timely diagnosis, individualized therapy, and a multidisciplinary approach are key to improving outcomes in patients with post-infarction left ventricular structural complications.
